# Assessment of the Vertical Dimension of Occlusion Using Palm Width and Finger Length

**DOI:** 10.3390/medicina60091526

**Published:** 2024-09-19

**Authors:** Cecilia Bacali, Mariana Constantiniuc, Antarinia Craciun, Daniela Popa

**Affiliations:** Department of Prosthodontics and Dental Materials, “Iuliu Hațieganu” University of Medicine and Pharmacy, 400006 Cluj-Napoca, Romania; cecilia.bacali@yahoo.com (C.B.); mconstantiniuc@umfcluj.ro (M.C.); popa.daniela@umfcluj.ro (D.P.)

**Keywords:** vertical dimension of occlusion, VDO assessment methods, palm width, finger length, VDO anthropometric methods

## Abstract

*Background and Objectives*: The vertical dimension of occlusion’s (VDO) assessment is a highly important issue in the everyday dentist’s practice. Patients with unstable occlusion, lost occlusal stops, extensive tooth loss in the lateral area, or complete edentulism need a proper assessment of the VDO before the prosthetic restoration is carried out. Subjective and objective methods were used over time for the restoration of VDO. The study aimed to investigate the possible correlation between finger length, palm width and the vertical dimension of occlusion. *Materials and Methods*: Assessment of the VDO for 236 subjects, Romanian and French dental students, was performed using the Willis Bite Gauge. The left hand of the subjects was scanned using a flat-bed scanner, and then measurements of palm width and finger length were carried out for each subject. Comparison between VDO values and finger length/palm width was conducted using one-way ANOVA and Student t-Test. *Results*: Higher VDO average values were found in French subjects compared with Romanian students. The same results were found according to gender; in both female and male subjects, lower values of VDO were found in the Romanian group. Higher values were obtained for women within each group when comparing to men. Statistically significant correlations of the analyzed parameters and VDO values were found. Higher statistical correlations of the studied variables were found for men compared to women in both groups. The highest statistical correlation was obtained between the VDO and the palm width measured at the fingerbase, followed by the middle finger length. *Conclusions*: The results showed the highest statistical correlation between the vertical dimension of occlusion and the palm width measured at the fingers’ base. Statistical correlations were also found between the VDO and the middle finger length. Simple formulas using finger length/palm width can be used for a rapid VDO determination.

## 1. Introduction

The Glossary of Prosthodontic Terms defines the vertical dimension (VD) as the distance between two selected anatomic or marked points (usually one on the tip of the nose and the other upon the chin), one on the fixed and one on the mobile jaw [1].

The vertical dimension of occlusion (VDO) is dependent on the presence of dental units and unaltered occlusal stops in the lateral areas of the arches. Unstable occlusion, lack of proper occlusal stops, absence of antagonist teeth (dental units) and complete edentulism, especially, can lead to difficulties or even the impossibility to directly measure the vertical dimension of occlusion. Errors in VDO registration are one of the main causes of denture treatment failure.

Alteration of the VDO (VDO increased or decreased) can alter esthetics of soft facial tissues and can induce speech difficulties, temporomandibular joint and craniomandibular disorders, muscle discomfort, and headache. On the other hand, craniomandibular disorders can affect chewing, swallowing, and breathing [2].

Determination of cranio-maxillo-mandibular relations is fundamental for a successful rehabilitation of totally or partially edentulous patients [3]. In order to restore the esthetics and dental maxillary functions using a denture, the clinician needs to accurately assess the ideal VDO of the patient [4].

Determination of the vertical dimension of occlusion (VDO) in edentulous patients is an essential step of the prosthetic treatment, along with correct recording of the denture bearing area, of the centric relation and adequate selection and placement of the artificial teeth [5]. 

Dentists often have difficulties in VDO evaluation [6] because of the large variations among individuals, hence the interest in finding the best method. Subjective methods of VDO assessment include the use of phonetics and esthetics, rest position of the mandible, and patient perceived comfort. Objective methods include the use of pre-extraction records, like intraoral measurements between different structures [7], photographs [8], cephalometric radiographs [9], phonation [10]. Electromyographic assessments and anthropological facial and body part measurements were also used for VDO restoration [2,11,12]. 

The anthropometric measurements are among the most accessible methods for VDO determination; different proportions between anatomic structures have been used along time to determine the vertical dimension of occlusion [4,5,13,14,15,16].

From old times, scientists like Marcus Vitruvius Pollio, Leonardo da Vinci, Leonardo Fibonacci searched for possibilities to replicate nature’s characteristics as close as possible, showing the marvelous relation between art and science [17,18,19]. More recent studies found that there is a relation between the anatomical dimensions in the human body structures due to the convergence of growth and development processes [20]. 

This study aimed to investigate the existence of a correlation between finger length, palm width, and the VDO. The null hypothesis was that the VDO cannot be correlated with the hand width or the finger length.

VDO values particularities in the studied group were also analyzed.

## 2. Material and Method

Permission for developing the study was obtained from the Ethics Committee of the University of Medicine and Pharmacy “Iuliu Hațieganu” Cluj-Napoca, Romania, before starting the research (DEP264/25.09.2023). The relevant aspects of the study were explained to each participant. All the subjects provided written consent for their participation in the study. The study took part in the Department of Prosthodontics, Faculty of Dental Medicine, University of Medicine and Pharmacy “Iuliu Hațieganu” Cluj-Napoca, Romania, and was addressed to students from 5th and 6th year, Romanian and French sections.

A total of 236 students who volunteered to participate in the study were randomly selected.

Inclusion criteria for the subjects were: right-handed subjects, intact and not deformed fingers, natural teeth without prosthetic restorations, lack of dento-maxillary anomalies, stable occlusion, facial symmetry. Exclusion criteria were: left-handed subjects, dento-maxillary anomalies, ongoing or previous prosthodontic or orthodontic treatments, bruxism, tooth loss in the lateral area, lost VDO, facial asymmetry, malocclusion.

**Figure 1:**
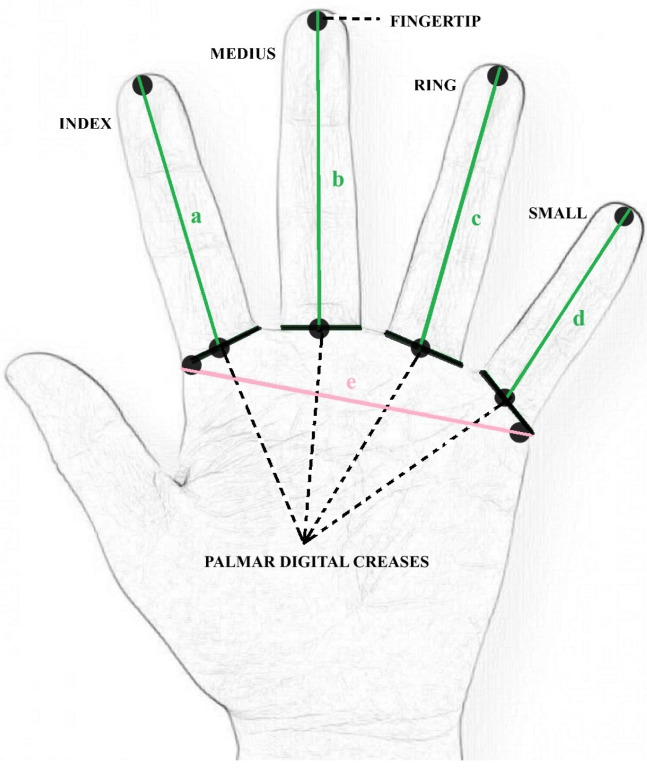
Graphic representation of measured finger lengths (a, b, c, d) and palm width (e).

The study consisted of measurements of the vertical dimension of occlusion, which was then compared with the left-hand width and the finger lengths (Figure 1). 

Two operators, teachers from the Department of Prosthetic of UMF “Iuliu Hatieganu” Cluj-Napoca, Romania, measured the VDO for each student. The measurements were conducted between the subnasale and gnathion points using the Willis Bite Gauge. The left hand of the subjects was scanned using a flat-bed scanner (HP Deskjet 3050 J 610 series) at 1200 dpi, and all the digital datasets were imported into the Image J software 1.54a.

After the left palm scanning, measurements (in mm) were performed using a ruler. Finger length was measured from the middle point of each palmar digital crease to the fingertip. Palm width was measured between the external points of the palmar digital creases, as shown in Figure 1.

Data were coded and gathered in Google Forms and Excel spreadsheet. The values for the VDO, finger lengths, and palm width were grouped (gender/nationality), and mean values and standard deviations were obtained. VDO values were analyzed considering nationality and gender as variables, and the correlation between the VDO and finger lengths/palm width was calculated within and between the groups using one-way ANOVA repeated measures and Student T-test. Statistical analysis was performed with GraphPad 6.01 (GraphPad Software, Inc.) statistical software. 

## 3. Results

Among a total of 236 dental students (63.6% Romanians and 36.4% French), 97 were males and 139 were females, with a maximum age of 53, a minimum age of 21, a mean age of 24,45, and a predominant age of 24.

### 3.1. VDO Assessment

**Figure 2:**
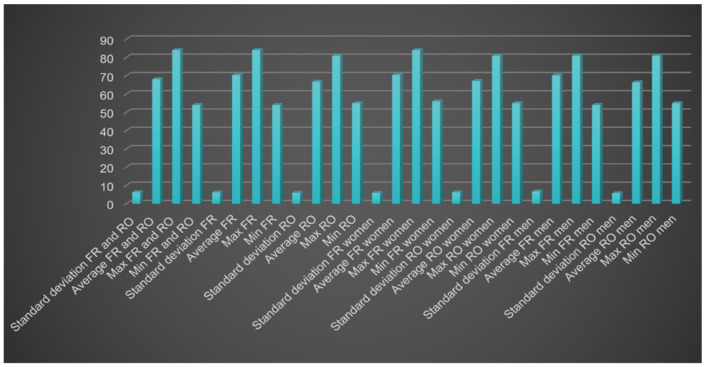
VDO analysis.

Analyzing the VDO (Figure 2, Table 1), the highest VDO value (84 mm) was registered in the French women group, and the lowest value (54 mm) was registered in the French men group.

In the Romanian group, a higher average value was recorded for women compared to men, with identical minimum and maximum values and a lower standard deviation for men. Analyzing the French group, a slightly higher average was recorded for women following the same pattern as in the Romanian group, with higher minimum and maximum values for women compared to men and a higher standard deviation for men.

Comparing Romanian and French groups, the values recorded for the French group were higher, for all the parameters studied besides the minimum value, without a significant difference. The same tendency was found when comparing Romanian and French women groups, with higher values besides the standard deviation for the French women group. Maximum values were recorded for Romanian and French men, with a higher average and standard deviation for French men, and a lower minimum value for French men. 

### 3.2. Comparison between VDO and Finger Length/Palm Width

**Figure 3:**
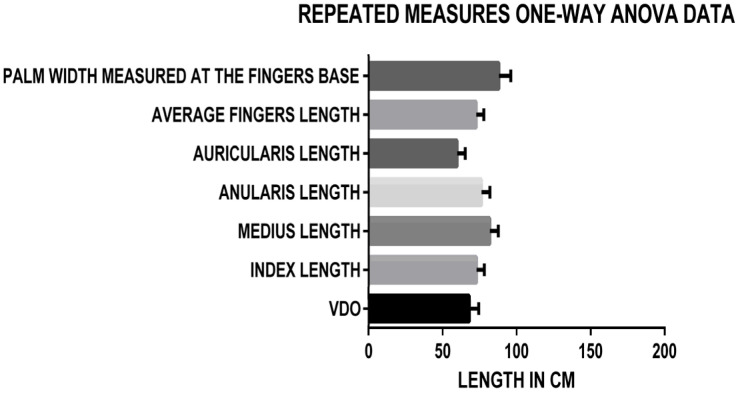
Repeated measures one-way ANOVA data Romanian and French, men and women.

The one-way ANOVA (Figure 3) showed statistically significant correlations (*p* < 0.0001) between all the parameters included in the study (average fingers length, palm width measured at the fingers base, index length, middle finger length, ring finger length—anularis Latin term, little finger length—auricularis Latin term) and the VDO for both the Romanian and French, men, and women groups, with an F value of 1497.

**Figure 4:**
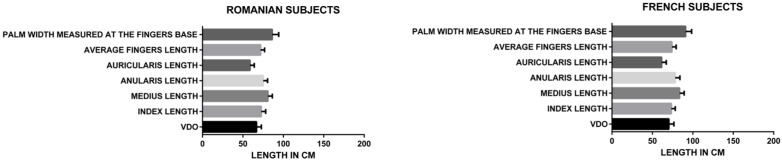
Repeated measures one-way ANOVA data Romanian and French subjects.

When analyzed separately, in the French (*p* < 0.0001) and Romanian groups (*p* < 0.0001) (Figure 4), the F value for the French group (716.4) was lower than for the Romanian group (857.9), demonstrating a higher statistical correlation of the analyzed parameters (average fingers length, palm width measured at the fingers base, index length, middle finger length, ring finger length, for the Romanian group.

**Figure 5:**
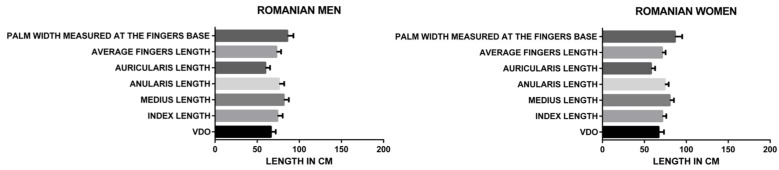
Repeated measures one-way ANOVA data Romanian men and women.

When analyzing separately women (*p* < 0.0001) and men (*p* < 0.0001) Romanian groups (Figure 5), the F value for the men group (501.8) was higher than for the women group (418.6), demonstrating a higher statistical correlation of the analyzed parameters (average finger length, palm width measured at the fingers base, index length, middle finger length, ring finger length, little finger length) for the men group.

**Figure 6:**
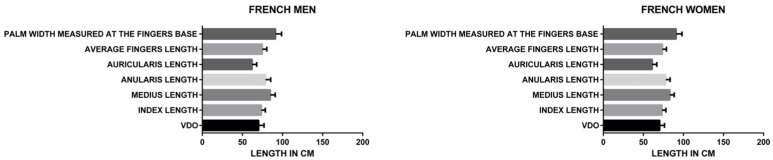
Repeated measures one-way ANOVA data French men and women.

When analyzing separately French and Romanian men groups (Figure 5 and Figure 6), the F value for the French group (220.6) was lower than for the Romanian group (501.8), demonstrating a higher statistical correlation of the analyzed parameters (average finger length, palm width measured at the fingers base, index length, middle finger length, ring finger length, little finger length) for the Romanian men group.

When analyzing separately French and Romanian women groups (Figure 5 and Figure 6), the F value for the French group (500.1) was lower than for the Romanian group (418.6), demonstrating a higher statistical correlation of the analyzed parameters (average finger length, palm width measured at the fingers base, index length, middle finger length, ring finger length, little finger length) for the French women group.

When analyzing separately women (*p* < 0.0001) and men (*p* < 0.0001) French groups (Figure 6), the F value for the men group (220.6) was higher than for the women group (500.1), demonstrating a higher statistical correlation of the analyzed parameters (average finger length, palm width measured at the finger base, index length, middle finger length, ring finger length, little finger length) for the women group.

**Figure 7:**
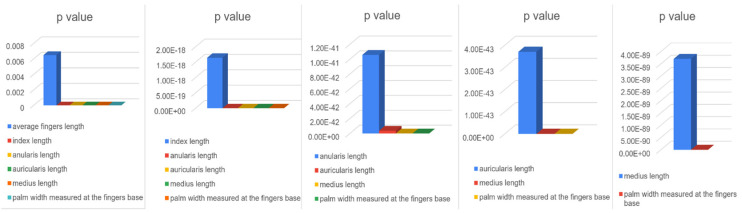
Student T-test statistical relevance.

**Figure 8:**
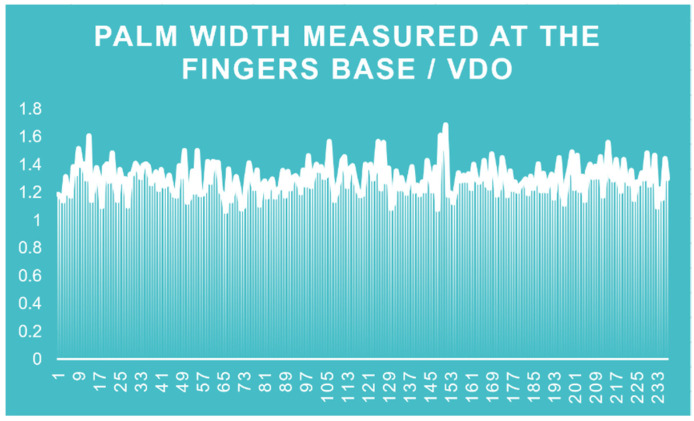
Palm width measured at the fingers base/VDO.

Using Student t-Test: Two-Sample Assuming Equal Variances to analyze the correlation between the VDO and all the other parameters investigated in the study for both Romanian and French, men and women groups, the following results (Figure 7) were found: average finger length (*p* = 0.006492359); palm width measured at the finger base (*p* = 6.2102 × 10^−111^); index length (*p* = 1.64289 × 10^−18^); middle finger length (*p* = 3.70399 × 10^−89^); ring finger length (*p* = 1.05908 × 10^−41^); little finger length (*p* = 3.70217 × 10^−43^), demonstrating the highest statistical correlation between the VDO and the palm width measured at the fingers base, followed by the middle finger length. Given these findings, the ratio between these two parameters and the VDO was analyzed, obtaining the following results:

1. VDO = 1.297530418 × W (Figure 8)

W = palm width measured at the finger base

average ratio (W/VDO) = 1.297530418; minimum ratio (W/VDO) = 1.064102564; maximum ratio (W/VDO) = 1.685185185.

**Figure 9:**
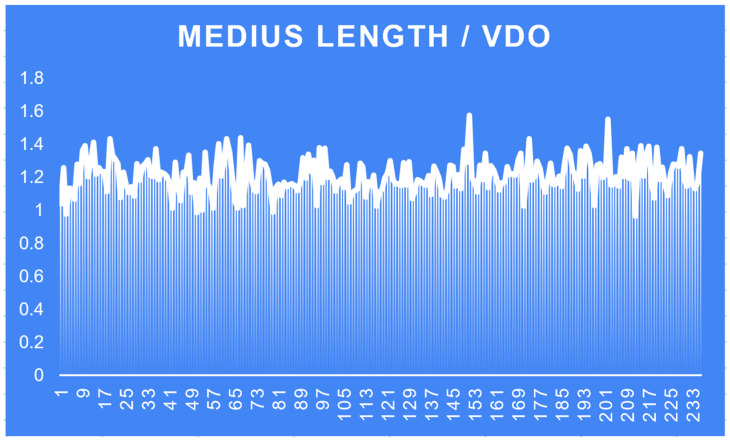
Middle finger length/VDO.

2. VDO = 1.210196594 × M (Figure 9)

M = middle finger length

average ratio (M/VDO) = 1.210196594; minimum (M/VDO) = 0.975; maximum (M/VDO) = 1.574074074.

## 4. Discussion

In the restoration of the dental maxillary apparatus, dentists aim to obtain not only the best functionality but also the best esthetics, the most natural appearance of the patient. For VDO determination, several methods using anthropometric measurements were proposed by different authors over time. The clinician needs to use the most accurate method to obtain the exact VDO of the patient for a successful prosthetic treatment.

Direct measurement with rulers or calipers is a simple method but may be susceptible to errors due to the soft tissue distortion [21]. Photographic analysis with or without facial reference markers has been used by some researchers [22]. Video analysis, proposed by Ackerman and Ackerman, is popular among orthodontic researchers [21]. Another method involves three-dimensional, noncontact imaging with laser or optical scanners [23,24]. Extensive research has been conducted concerning age-related changes in the smile [25]. With increased age, the inter-labial gap height (the vertical distance between the upper and lower lips) decreases, the inter-commissural width (the distance between the left and right commissures) decreases, and the smile index (width-height) increases [21]. 

The evaluation is influenced by operator and patient variables such as mental and physical state, utilization of different measurements or tools, and expertise of the operator [13]. Failure to record the optimum vertical dimension can lead to functional and esthetic complications such as cheek biting, difficulty swallowing, decreased lower facial height, pain in the TMJ area, angular cheilitis, loss of lip fullness, and Costen’s syndrome [13]. Each method possesses certain inherent disadvantages, such as higher cost, longer time, difficulty in application, and errors in measurement. 

The method used in this study is an objective method, easy, inexpensive, and possible to be carried out in a short time without requiring complex devices. 

The mean values of the vertical dimension of occlusion as shown in the literature vary between 64 mm and 73 mm for males and 65 mm to 69 mm in females [13]. In our study, the values recorded vary between 54 cm and 84 cm. In the Romanian group, the minimum value of VDO was 55 cm and the maximum was 81 cm. In the French group, the minimum value of VDO was 54 cm and the maximum 84 cm.

Analyzing the statistical results that were obtained, significant differences were found between the group of Romanian students (the average VDO 66.8) and those of French speakers (the average VDO 70.36).

In the Romanian student group, the average VDO was lower by 3.56 mm than the average of the values identified in the other studied group.

The same difference in the average values of VDO was found in the groups of men and women, being in favor of the French-speaking population. The difference between the two groups of students can be a consequence of the fact that the Romanian population is more uniform and originated from the same area of the country, while the group of French-speaking students includes both French, Algerian, or Tunisian subjects.

Similar studies, which were based on finger length measurements, obtained different conclusions, and the conflicts in results were attributed to the variations in gender and race [4,14,15].

A study from 2019 concluded that index finger length is almost equal to VDO and may be useful in VDO determination [26]. Khan et al. studied the relations between the vertical dimension of occlusion and the length of the index finger of the right hand, finding significant statistical correlations in both genders [27]. The current research identified the highest statistical correlation between the length of the middle finger and the VDO, although significant correlations between the index finger length and the VDO were also found. 

The mean recorded values of the index finger length in the present study were 71.46 mm in the Romanian women group 73.26 mm in the French women group, 74.19 mm in the Romanian men group, and, respectively, 73.63 mm in the French men group. Other studies report similar results or slightly lower values, as follows: 65.9 mm in females, 71.6 mm in males [15]; 73.54 mm in males and 69.95 mm in females [28]; 64.9 mm in males and 65.2 mm in females [29]; 72.9 mm in males and 66.9 mm in females [30]. The present research also explored racial differences and found variations in finger length among different racial groups (Romanian and French). The difference between the values of the above-mentioned studies and the present research can be explained by considering the same statement.

Other authors [16] did not find correlations between finger length and different facial measurements.

This study analyzed the left hand, as it was supposed that the right hand, being more used in different activities, can be hypertrophied or can show changed dimensions of certain fingers. Additionally, some studies have found no differences in finger length between the two hands [28].

Another study has shown that sexual dimorphism is present, with men having greater values for VDO and longer finger lengths compared to women. This difference in finger length is associated with levels of androgen exposure after puberty [31]. The present research revealed similar results in both groups analyzed (Romanian and French).

The inclusion of young patients in the study presents the advantage that the bone system is not affected by the inherent changes due to aging. In this study, we assumed that VDO is preserved as it is the length of the left-hand fingers.

Among the limitations of the study are the limited number of participants and of ethnic groups that were included in the research, which can influence the sample’s variety. Other confounding factors like age, dentomaxillary anomalies, prosthetic restorations, missing teeth, attrition, and facial asymmetry might influence the result and could also have been considered. Further studies could be conducted for these issues.

This study suggests that the calculation of the VDO based on the proposed formulas can be used in the restoration of the facial lower height as a complementary method, which can be performed in a short time without special equipment.

## 5. Conclusions

Higher VDO average values were found in French subjects comparing with Romanian students, and also higher statistical correlations of the studied variables were found for men comparing to women in both groups.

The study demonstrates the highest statistical correlation between the VDO and the palm width measured at the finger base, followed by the middle finger length. An innovative method with practical value, based on easy and rapid measurement and calculation (using simple formulas), was proposed for VDO determination.

## Figures and Tables

**Table 1:** Vertical dimension of occlusion (VDO) values.

	VDO Average (mm)	Min (mm)	Max (mm)	Standard Deviation (mm)
Romanian and French Men and Women	68.09	54	84	6.31
Romanian Men and Women	66.8	55	81	6.02
French Men and Women	70.36	54	84	6.20
Romanian Women	67.17	55	81	6.27
French Women	70.42	56	84	5.96
Romanian Men	66.35	55	81	5.72
French Men	70.23	54	81	6.73

## Data Availability

The data supporting the findings of the study are available from the corresponding author upon reasonable request.
